# Why oppositely charged ions of equal radii have different heats of hydration?

**DOI:** 10.1007/s40828-017-0045-9

**Published:** 2017-05-02

**Authors:** Jan S. Jaworski

**Affiliations:** Faculty of Chemistry, University of Warsaw, Pasteur Str. 1, 02-093 Warsaw, Poland

**Keywords:** Ion hydration, Alkali metal cations, Halide anions, Born equation, Electrostatic interactions, Hydrogen bonds

## Abstract

Looking for the answer to the title question a number of oversimplifications of the Born model of ion hydration are discussed. They involved: ionic radius, dielectric saturation, structure of water molecules around ions and the nature of ion–water interactions. On the basis of recent literature the last factor—pure electrostatic interactions of alkali metal cations with water molecules but hydrogen bonding of halide anions—has been found to decide on the minimum energy of interactions, the charge transferred between interacting species in equilibrium and the distance between them. Thus, different nature of interactions for cations and anions explains difference in their hydration heats as well as the observation that solvent–solvent interactions in hydrogen bond donor solvents give the important contribution to solvation heats only for anions.

## Introduction

The enthalpy of hydration of an ion $\Delta H_{{{\text{i}},{\text{hydr}}}}^{^\circ }$ (and the corresponding Gibbs free energy $\Delta G_{{{\text{i}},{\text{hydr}}}}^{^\circ }$) plays an important role in the elucidation of a behavior of ions in aqueous solutions including thermodynamic as well as kinetic aspects of a number of ionic reactions considered in courses of general and inorganic chemistry. Usually the starting point for most considerations on the solvation of ions is the familiar Born equation [1] which describes the electrostatic work due to growing polarization of a medium [2] when one mol of a spherical ions of radius *r*_i_ and charge *z*_i_ *e*_o_ is transferred from a vacuum of permittivity *ε*_o_ into a solvent treated as a continuum dielectric medium with a relative permittivity of *ε*_s_:

1 $$\Delta G_{{{\text{i}},{\text{hydr}}}}^{^\circ } = \, - \, (N_{\text{A}} z_{\text{i}}^{ 2} e_{\text{o}}^{ 2} / 8\pi \varepsilon_{\text{o}} r_{\text{i}} ){ (1 }{-}{ 1}/\varepsilon_{\text{s}} ) ,$$

where *N*_A_ is Avogadro’s constant. The enthalpy of solvation is given by the similar Born–Bjerrum equation [3] but with the additional term involving the temperature derivative of the solvent permittivity:

2 $$\Delta H_{{{\text{i}},{\text{hydr}}}}^{^\circ } = \, - \, (N_{\text{A}} z_{\text{i}}^{2} e_{\text{o}}^{2} / 8\pi \varepsilon_{\text{o}} r_{\text{i}} ) \, \left[ { 1 { }{-}{ 1}/\varepsilon_{\text{s}} - T/\varepsilon_{\text{s}}^{2} \left( {{\text{d}}\varepsilon_{\text{s}} /{\text{d}}T} \right)} \right].$$

**Fig. 1:**
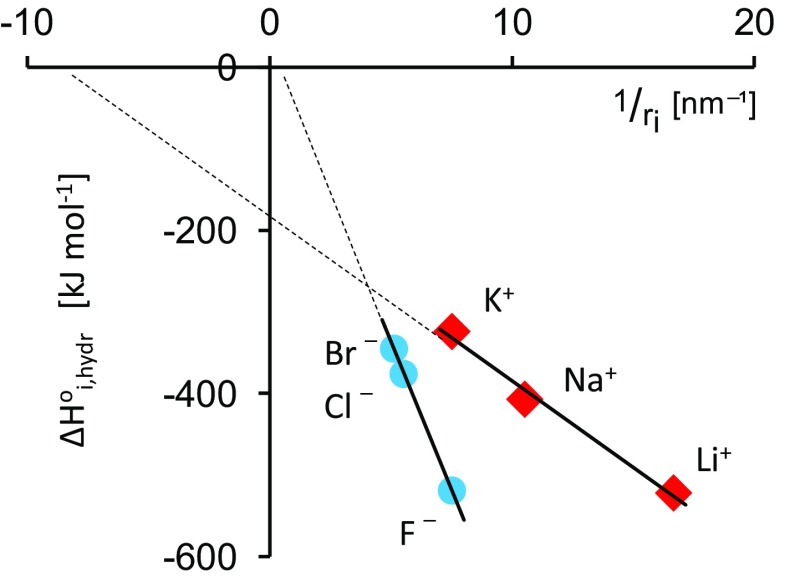
Relationships between the heat of hydration, $\Delta H_{{{\text{i}},{\text{hydr}}}}^{^\circ }$, [4] and the reciprocal of ionic radius [5] for monatomic cations and anions

Qualitatively Eqs. (1) and (2) predict correctly higher negative values of $\Delta G_{\text{i,hydr}}^{^\circ }$ and $\Delta H_{\text{i,hydr}}^{^\circ }$ for smaller ions with higher charges and in solvents with a higher permittivity. However, a quantitative comparison between experimental and computed values are not satisfactory as was discussed repeatedly by many authors for aqueous solutions of monatomic and univalent ions (in particular alkali metal cations and halide anions for which spherical shape is most adequate). The enthalpy of hydration which does not include the entropy term and can be directly compared with results of some theoretical calculations will be considered here. In general, three problems arise then. The first is that the Born estimates of $\Delta H_{{{\text{i}},{\text{hydr}}}}^{^\circ }$ are too large in magnitude than absolute values obtained from experimental data with some extrathermodynamic assumption [4] and the differences observed are greater for cations than for anions as shown in Table 1. Second, for cations and anions of the same size Eq. (2) predicts the same value of $\Delta H_{{{\text{i}},{\text{hydr}}}}^{^\circ }$. However, more negative values are observed for anions (Table 1), e.g., for K^+^ and F^−^ ions for which Pauling’s crystal radii [5] are similar (133 pm) as well as for Na^+^ (*r*_i_ = 116 pm) and F^−^ (*r*_i_ = 119 pm) if crystal radii of Shannon and Prewitt based on electron density measurements [6] are used. Third, Eq. (2) requires that the intercept of the linear plot of $\Delta H_{{{\text{i}},{\text{hydr}}}}^{^\circ }$ against 1/*r*_i_ is equal to zero but it is not the case, in particular for cations (Fig. 1). A number of corrections to the original Born equation were repeatedly proposed and often they are mentioned in modern textbooks but some of them have only historical meaning and it is hard to choose which effect is mainly responsible for the title question.

**Table 1:** Standard molar enthalpies of hydration of ions at 298 K [4] absolute^*a*^ and calculated from Eq. (2)

$- \Delta H_{{{\text{i}},{\text{hydr}}}}^{^\circ }$/kJ mol^−1^		
Ion	Expt	Born^b^
Li^+^	522	1163
Na^+^	407	735
K^+^	324	524
F^−^	519	524
Cl^−^	376	386

^a^Obtained from conventional values assuming for the hydrogen ion $\Delta H_{{{\text{i}},{\text{hydr}}}}^{\text{o}}$ = −1094 kJ mol^−1^

^b^Calculated using Pauling’s ionic radii [5]

In general, it is clear that the Born model cannot give the correct results because of its oversimplifications: ions are not rigid spheres with the same radius as in crystals, solvent is not continuum dielectric medium but has a molecular structure, solvent electric permittivity decreases dramatically in the strong electric field near an ion, and finally solvent–ion interactions have different nature depending on chemical properties of a given system and cannot be always limited to pure electrostatic interactions. They all will be briefly discussed below.

## Ionic radius and disruption of water structure

The use of the effective ion radius *r*_eff_ = *r*_i_ + *δ*_s_ in the Born equation, i.e., increasing Pauling’s radius of cations *r*_i_ in aqueous solutions by *δ*_s_ ≈ 80 pm, results in a correct plot of $\Delta H_{{{\text{i}},{\text{hydr}}}}^{^\circ }$ against (*r*_i_ + *δ*_s_)^−1^ with the intercept equal to zero [7]. The correction term *δ*_s_ is usually found by fitting experimental data although it can be calculated in some simple models of solutions [8] and can be related to results of statistical mechanical [9] and molecular dynamics [10] simulations. The *δ*_s_ term is different for cations and anions and depends on the solvent nature. It is interesting to note that a recent comparison of experimental $\Delta G_{{{\text{i}},{\text{solv}}}}^{^\circ }$ values for monatomic ions in 17 solvents using *r*_eff_ = *r*_i_ + *δ*_s_ with Pauling radii *r*_i_ showed [8] that the *δ*_s_ term depends on solvent Lewis basicity and Lewis acidity for cations and anions, respectively. Thus, it reflects specific, chemical ion–solvent interactions.

On the other hand, the idea of adding the *δ*_s_ term to *r*_i_ in the Born equation is usually explained in terms of increasing the ionic radius to account for the disruption of solvent structure around this ion [11]. Such procedure reduces the negative Gibbs energy and enthalpy of an ion according to Eqs. (1) and (2) by the energy which is necessary for changing dipolar solvent–solvent interactions around an ion and in particular, hydrogen bonds between water molecules. It clearly explains a discrepancy between experimental and calculated values for each ion given in Table 1. Moreover, higher *δ*_s_ values for cations than for anions (confirmed in recent analysis for monatomic ions [11, 12]) can explain smaller hydration of K^+^ ion than F^−^ (Table 1) due to a stronger breaking of water structure around the cation [11]. Thus, the further discussion of the effective ionic radius should take into account the breaking of the original water structure by some ions as first noted by Bernal and Fowler [13] and explicitly described by Frank and Evans [14, 15]. The last authors proposed the model of water structure in aqueous solutions consisting of three concentric layers around an ion: the innermost layer with water molecules strongly oriented to an ion, the second region with the broken original water structure, and the third one with the original H-bonded structure of water molecules a little polarized by the relatively weak ion field at larger distance from an ion. The relative extension of the second layer depends on the ion nature (charge and size) and this layer can dominate in observed properties. Different structure of broken regions for cations and anions formed as a monolayer outside the first coordination shell was also considered by Bockris with coworkers [16]: for cations, it consists of water monomers some of which liberate with the respect to molecules in the first shell while for anions hydrogen bonds occur between water molecules in the first shell and in the structure broken region.

The classic suggestion of Frank and Evans [14, 15] is in agreement with experimental parameters proposed later by other authors to determine quantitatively the structure-making or structure-breaking character of ions in aqueous solutions. Such parameters based on the activation energy of water exchange caused by the ion, the change of ion entropy, the effect of ions on viscosity of water and the difference of solubility of salts in light and heavy water have been recently tabulated by Marcus [17] with references to original papers. They all indicate that small ions (Li^+^, F^−^) are structure-making ions, K^+^ ion is slightly structure-breaking and larger ions (Rb^+^, Cs^+^, Br^−^, I^−^) are evidently structure-breaking [14, 15, 17]. Thus, the opposite behavior of K^+^ and F^−^ ions is in accordance with the difference in their $\Delta H_{{{\text{i}},{\text{hydr}}}}^{^\circ }$ values discussed above. However, it is not the case for other monatomic ions for which the size determines mainly their effect on water structure and not the sign of the charge. Thus, the assumption that smaller negative values of $\Delta H_{{{\text{i}},{\text{hydr}}}}^{^\circ }$ for cations than those for anions are caused by stronger disruption of original water structure by positive ions is not correct and another explanation should be considered.

## Dielectric saturation

The enormous gradient of the electrostatic potential near the surface of an ion causes the strong polarization of a solvent. It results in an extreme decrease of the relative permittivity *ε*_s_ near the ion (as reviewed in [18]) or in a more realistic model [19] the gradual decrease of *ε*_s_ in a series of concentric spherical layers around ion, each with a different relative permittivity. The smaller value of local permittivity used in the Born equation results in smaller $\Delta H_{{{\text{i}},{\text{hydr}}}}^{^\circ }$ values in agreement with experimental data. Assuming that a discrepancy between calculated and experimental values of thermodynamic functions of ions depends only on dielectric saturation, Noyes obtained [20] effective dielectric constants which were very small and of course higher for anions than for cations. However, for cations having the electronic structure of an inert gas and charge numbers of 1, 2 and 3 he found [20] that effective dielectric constants depend only on size of an ion but are virtually independent of charge. All other models describing variation of medium relative permittivity with the distance from an ion [18, 21] assumed as well the independence of the magnitude and sign of a charge. Thus, the effect of dielectric saturation cannot be responsible for differences between properties of cations and anions in aqueous solutions.

## Structural aspects of aqueous solutions

Different arrangements of a water molecule interacting with a cation and with an anion were pointed out in the literature and their discussion is related to various kinds of possible interactions [18]. In general, interactions of a cation with a lone pair at water oxygen atom analogous to H-bonding of an anion were considered [22] or simple electrostatic interactions between an ion and a point-dipole [13, 23] or quadrupole [24, 25] of water molecule. Concerning ion–dipole interactions Bernal and Fowler noted [13] that the dipole moment of water molecule is not centrally distributed between three atoms and thus, the positive end of a dipole can get closer to anions than the negative end can to cations. That explains experimentally observed stronger hydration of anions as cited by other authors [20, 23, 26]. Modern X-ray diffraction studies of aqueous KF solutions [27, 28] indeed supported a shorter ion–oxygen distance for F^−^ anions (262 pm) than for K^+^ cations (295 pm) and of course much shorter anion–deuterium distance, e.g., 222–226 pm for Cl^−^–D as obtained from neutron diffraction measurements in LiCl and NaCl solutions [28, 29]. However, distances between interacting species should be rather understood as consequences of the nature and energy of interactions as will be discussed later. The equilibrium distance depends on the minimum of interaction energy which in turn corresponds to a compromise between attractive and repulsive forces. On the other hand, Buckingham considering water molecules as electrical quadrupoles found [24, 25] a positive contribution to hydration heats for cations but negative for anions due to different orientations of water molecules towards both kinds of ions. The above result explains more negative experimental $\Delta H_{{{\text{i}},{\text{hydr}}}}^{^\circ }$ values for anions.

**Fig. 2:**
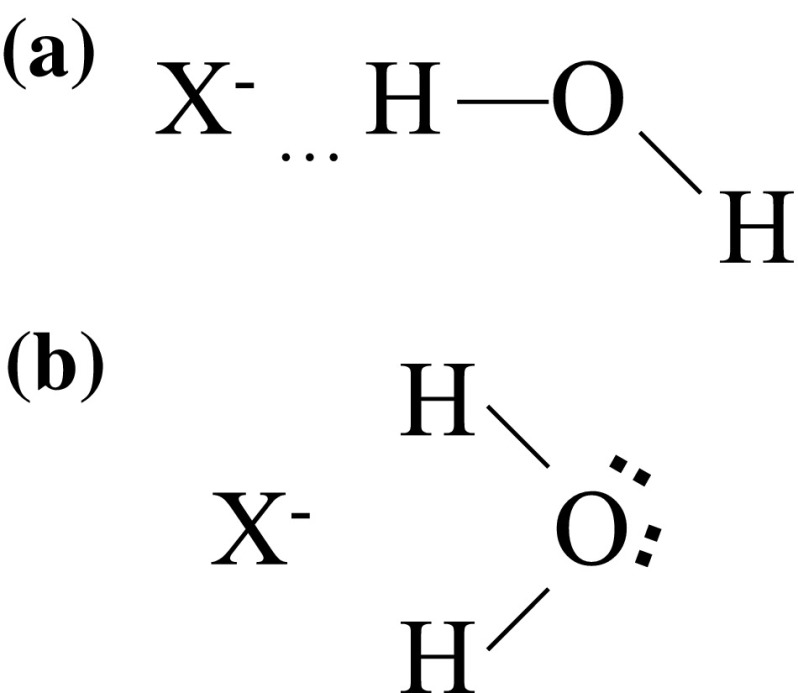
Two possible planar orientations of water about anions: **a** linear H-bonded [13]; **b** normal ion–dipole and ion-quadrupole [24, 25]

Nevertheless, the above discussions were restricted to pure electrostatic interactions and cannot explain correctly the title question if interactions have some chemical nature. Bernal and Fowler assumed [13] the planar structure of an anion–H_2_O entity, in which an anion is located in the same line as the H–O bond favorable to hydrogen bonding (Fig. 2a). On the other hand, Buckingham suggested [24, 25] that an anion is located between both hydrogen atoms (the symmetry *C*_2v_ as in Fig. 2b). Thus, the formation of hydrogen bonds is not possible and interactions of an anion with H_2_O molecules are essentially ion-dipolar and ion-quadrupolar. Similar symmetric orientations of a water quadrupole to ions were also discussed recently by Bockris and Reddy [30]. However, neutron diffraction studies of aqueous solutions of alkali metals chlorides [28, 29] as well as X-ray diffraction and infrared spectroscopy investigations [28] of solutions of other halides (cf., examples in [31]) evidently support the structure shown in Fig. 2a for all halide ions in aqueous solutions. For example, the deviation of the Cl^−^–D–O angle from 180° is negligibly small [28, 29], close to 0° for LiCl solutions [29]. Thus, dipolar and quadrupole models look unlikely and hydrogen bonding to anions should be taken into account as the primary factor causing different behavior of cations and anions.

## Nature of ion–water interactions

The electrostatic nature of interactions between water molecules and alkali metal cations, in particular hard [32] or nonpolarizable small cations, looks quite acceptable and can be confirmed by recent quantum-chemical calculations [33]. However, for anions the role of hydrogen bonding was well recognized in modern literature [4, 11, 31] and supported by neutron and X-ray diffraction studies [27, 28, 31]. The above different nature of interactions was mentioned in some discussions on difference in $\Delta G_{{{\text{i}},{\text{hydr}}}}^{^\circ }$ and $\Delta H_{{{\text{i}},{\text{hydr}}}}^{^\circ }$ values for cations and anions of the same size but only Sharpe [31] explicitly stated that it “is very probably a major reason for this, but other factors also appear to be involved”.

Before further discussion, it will be worthy to remember that for monatomic cations with charge numbers of 1, 2 and 3, the function [1 − ($\Delta H_{{{\text{i}},{\text{hydr}}}}^{^\circ }$)_exp_/($\Delta H_{{{\text{i}},{\text{hydr}}}}^{^\circ }$)_Born_] of the ratio of experimental and calculated hydration enthalpy decreases with increasing ionic radius forming a reasonable curve [20]. However, substantial deviations to lower values were observed for halide anions as well as for Hg^2+^, Cu^+^ and Ag^+^ cations. Extremely high experimental $\Delta H_{{{\text{i}},{\text{hydr}}}}^{^\circ }$ values for last two cations (e.g., for Cu^+^ $\Delta H_{{{\text{i}},{\text{hydr}}}}^{^\circ }$ = −535 kJ mol^−1^ [4] whereas for Na^+^ with a similar size $\Delta H_{{{\text{i}},{\text{hydr}}}}^{^\circ }$ = −375 kJ mol^−1^) were difficult for explanation. However, at present they can be easily related to more covalent interactions of soft cations with water molecules, e.g., for Ag^+^ ion ($\Delta H_{{{\text{i}},{\text{hydr}}}}^{^\circ }$ = −440 kJ mol^−1^) as compared with smaller Na^+^ ion [34]. The same explanation can be also proposed here for anions. The hydrogen bonding of anions to water molecules in aqueous solutions means the covalent nature of their interactions which are stronger and result in more negative values of $\Delta H_{{{\text{i}},{\text{hydr}}}}^{^\circ }$ than those for alkali metal cations of similar size.

**Fig. 3:**
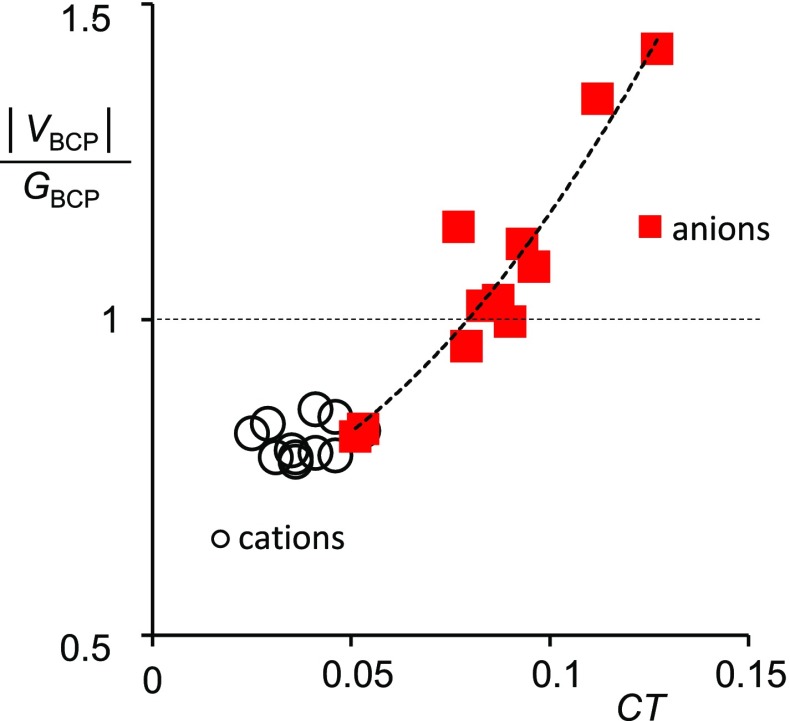
Covalent character of interactions given by the ratio of |*V*_BCP_|/*G*_BCP_ plotted against charge transferred, CT, from lone electron pairs at O or N atoms in solvent molecules to cations or from anions to HO- or HN- groups in solvent molecules. Data from Reference [33]

Different nature of interactions between monatomic cations and anions under consideration was evidently shown in our recent theoretical calculations [33]. The formation of complexes between solvent molecule and ion was considered there for three hard cations: Li^+^, Na^+^ and K^+^, which interact with lone electron pairs of O or N atoms of hydrogen bond donor (HBD) solvents (in particular water, methanol, formamide, and ammonia for which experimental $\Delta H_{{{\text{i}},{\text{solv}}}}^{^\circ }$ values are known [4, 35]) and for three anions: F^−^, Cl^−^, Br^−^, which form hydrogen bonds with the same solvents. The total energy of interaction, *E*_total_, and the amount of charge, CT, which is transferred in the complex formed were calculated. Moreover, to characterize the nature of interactions the ratio of potential to kinetic electron energy density at bond critical point |*V*_BCP_|/*G*_BCP_ [36] was calculated using the quantum theory of atoms in molecules (QTAIM). The increase in the |*V*_BCP_|/*G*_BCP_ ratio indicates a more covalent character of the bond. For interactions of solvent molecules with cations small values of charge transferred to cations were found [33] and the ratio |*V*_BCP_|/*G*_BCP_ <1 as shown in Fig. 3. The above results indicate a pure closed-shell type of interactions. Thus, predominant role of electrostatic interactions between alkali metal cations and solvent molecules was supported [33]. On the other hand, for anions the positive values of the Laplacian in combination with larger values of the |*V*_BCP_|/*G*_BCP_ (mostly >1 with the exception of Cl^−^ and Br^−^ ions in ammonia [33], as shown in Fig. 3) indicate a partially covalent character of interactions in equilibrium H-bonded complexes. It is also evident from Fig. 3 that for anions the values of charge transferred, CT, are higher than those for cations resulting in stronger bonds with solvent molecules in equilibrium complexes. Thus, distances *d* between ions and interacting atoms of solvent molecules are shorter. For example, a comparison of oppositely charged ions of similar Pauling radius showed for the complex of water molecule with K^+^ ion: *CT* = 0.025 a.u., *E*_total_ = −76.8 kJ mol^−1^ and the O···K^+^ distance *d* = 261.8 pm, whereas for the complex with F^−^ ion: *CT* = −0.112 a.u. (negative charge is transferred in the opposite direction, i.e., from the anion to the solvent molecule), *E*_total_ = −111.0 kJ mol^−1^ and the H···F^−^ distance *d* = 139.9 pm [33].

Interaction energies calculated are in good agreement with experimental data supporting a correctness of the proposed model. Namely, for three cations in four solvents a linear correlation was found [33] between *E*_total_ and experimental $\Delta H_{{{\text{i}},{\text{solv}}}}^{^\circ }$ values [4]:

3 $$E_{\text{total}} = \, 0. 40\; (\pm 0.0 4 )\Delta H_{{{\text{i}},{\text{solv}}}}^{^\circ } + 60\;( \pm 1 6),$$

which holds for *n* = 12 points with the square of the correlation coefficient *R* ^2^ = 0.984. On the other hand, points for anions (*n* = 11) deviate from the above correlation line. However, they could be described by two-parameter dependence including $\Delta H_{{{\text{i}},{\text{solv}}}}^{^\circ }$ and the molar heat of vaporization for a given solvent, Δ*H*_vap_:

4 $$E_{\text{total}} = \, 0. 4 2\; (\pm 0.0 7 4 )\Delta H_{{{\text{i}},{\text{solv}}}}^{^\circ } {-}{ 1}. 2\; (\pm 0. 3 )\Delta H_{\text{vap}} + { 148}\; (\pm 2 9 ) ,$$

which holds for *R* ^2^ = 0.976 and the addition of the second parameter Δ*H*_vap_ is statistically important with the probability of 99.73% [33]. Equation (4) indicates that solvent–solvent interactions are important in solvation heats of anions by water and other HBD solvents but not for cations.

The last result is in accordance with earlier analysis of Fawcett who modified the Born equation by multiplying Eq. (1) by the *f*_dd_ term which describes the effect of dipole–dipole interactions and H-bonding between solvent molecules on $\Delta G_{{{\text{i}},{\text{hydr}}}}^{{_{^\circ } }}$ values and found that for monatomic ions in aqueous solutions the *f*_dd_ term is important only for anions [12, 37]. The following simple explanation of these different behaviors of cations and anions can be proposed: the formation of hydrogen bonds which have covalent character needs a substantial rearrangement of solvent structure around an anion because these bonds are directional, whereas similar significant rearrangements are not necessary for pure electrostatic, not directional interactions of water molecules with alkali metal cations.
